# Application of exercise therapy in patients with chronic kidney disease-induced muscle atrophy: a scoping review

**DOI:** 10.1186/s13102-024-00876-8

**Published:** 2024-04-30

**Authors:** Jiawei Yin, Xiaotu Zhang, Zilin Wang, Zihan Qu, Xuefeng Sun, Yuqing Song, Hongshi Zhang

**Affiliations:** Changchun University of Chinese Medicine, No.1035, Boshuo Road, Jingyue Development District, Changchun, 130117 China

**Keywords:** Chronic kidney disease (CKD), Muscle atrophy, Sarcopenia, Exercise Therapy, Scoping review

## Abstract

**Background:**

The prevalence of muscle atrophy in patients suffering from chronic kidney disease (CKD) presents a significant challenge to healthcare providers, necessitating innovative approaches to management and care. Against this backdrop, this study embarks on a comprehensive review of literature concerning the application of exercise interventions in the nursing care of these patients. Such interventions are critical in addressing the debilitating effects of the condition, which include progressive loss of muscle mass and strength, adversely affecting patient mobility, quality of life, and overall survival. This review aims to identify the specific exercise modalities, contents, outcome indicators, and application effects associated with this intervention, in the context of the complex interplay of metabolic, inflammatory, and hormonal factors contributing to muscle wasting in CKD patients. By examining the efficacy of various exercise interventions, this study seeks to elucidate optimal strategies for mitigating the impact of CKD-induced muscle atrophy, thereby informing clinical practices and improving patient outcomes.

**Methods:**

According to the method of a scoping review, nine databases (Cochrane, PubMed, EMBASE, Web of Science, ProQuest, Ovid, CNKI, Wanfang Data, and VIP) were searched until September 28, 2023. The included literature was screened, summarized, and analyzed.

**Results:**

A total of 20 pieces of literature were included. Some types include aerobic exercise, resistance exercise, and aerobic combined resistance exercise. The exercise intensity primarily falls within the mild to moderate range, with a recommended frequency of 2 − 3 times a week, lasting 30 − 60 min each time. The types of outcomes encompassed in this study include body composition, functional testing, strength measurements, laboratory examinations, cardiopulmonary function assessments, and patient-reported outcomes. To varying degrees, exercise intervention positively impacts the subjects' physical activity ability, body composition, and skeletal muscle status. Currently, resistance training is the primary type of intervention used for preventing and treating CKD patients induced by muscle atrophy.

**Conclusion:**

Exercise intervention can improve muscle strength, physical function, and quality of life in patients with CKD muscle atrophy. Therefore, patients should be fully informed of the effect of exercise intervention in the treatment of chronic kidney disease-induced muscle atrophy in future, so as to promote the standardized implementation of exercise intervention.

**Supplementary Information:**

The online version contains supplementary material available at 10.1186/s13102-024-00876-8.

## Introduction

Chronic kidney disease (CKD) significantly impacts global health, with its management complicated by the high prevalence of sarcopenia, identified as a loss of skeletal muscle mass and strength. Duarte et al. [1] report a notable prevalence of sarcopenia in CKD patients at 24.5%, with a higher incidence observed in those undergoing dialysis. This condition, especially severe in 26.2% of dialysis patients compared to 3.0% in non-dialysis patients, underscores the critical need for early identification and intervention.Chronic Kidney Disease (CKD) is associated with various pathophysiological processes, including mitochondrial dysfunction. Studies reported that muscle atrophy in CKD patients is related to mitochondrial dysfunction [2]. Exercise intervention can reduce oxidative stress, increase mitochondrial capacity, and enhance mitochondrial function [3]. Furthermore, it is particularly noteworthy that research has identified the combination of moderate-intensity continuous exercise (MICE) with blood flow restriction (BFR) as effective in suppressing the appetite of obese adults through the promotion of Lac-Phe and ghrelin secretion [4]. This finding underscores the significant implications of exercise not only for CKD but also for diabetes management, highlighting its potential as a pivotal intervention in the treatment and prevention strategies for these conditions. Mitochondrial DNA copy number (mtDNA-CN) is considered a novel biomarker for CKD risk, as higher levels of mtDNA-CN are associated with a lower risk of CKD. This relationship is independent of traditional CKD risk factors, suggesting that mtDNA-CN could serve as an important indicator for identifying CKD progression and severity [5]. Furthermore, the onset of diabetes in patients with CKD is significant, as mitochondrial dysfunction can exacerbate metabolic dysregulation. The relationship between mitochondrial function and CKD highlights the potential for targeted interventions to improve mitochondrial health and manage CKD, particularly in the context of comorbid conditions like diabetes [6].

Recent meta-analyses showed that exercise training can increase cardiorespiratory endurance, improve muscle strength and muscle volume, reduce the risk of cardiovascular disease, delay the progression of CKD, and improve the quality of life of CKD patients [7]. Therefore, we should identify muscle atrophy in CKD patients as early as possible and implement the intervention. While patients diagnosed with CKD may participate in resistance training, flexibility exercises, and aerobic exercise [8], it is crucial to consider the diverse physical capacities and tolerances of subjects at various phases of CKD. Furthermore, the lack of clear evaluation criteria for the type, specific content, and outcomes of exercise interventions for CKD patients leads to significant heterogeneity in the type, frequency, and intensity of exercise interventions [7].

A scoping review assists researchers in elucidating their research inquiries, presenting the scope and extent of research involved, summarizing research results, identifying the limitations of existing research, and finding research progress in a particular knowledge field [9]. This study used a scoping review methodology to analyze and synthesize the application research on exercise intervention in chronic nephritis muscle atrophy. Our objective is to furnish medical and nursing professionals with a comprehensive summary of exercise intervention types, content elements, evaluation indices, and efficacy, serving as a valuable resource.

## Methods

### Research question

① What are the methods, intensity, frequency, and time of exercise intervention for chronic kidney disease-related muscle atrophy? ② How does the application affect the exercise intervention program?

### Identifying relevant studies

#### Search strategy

Using a computer, a search was conducted in the Cochrane Library, PubMed, EMBASE, Ovid, ProQuest, Web of Science, CNKI, Wanfang, and VIP. The search time was covered from the establishment of the database until September 2023. The search words were combined with the MeSH words and entry terms in the PubMed database, including the search terms "Sarcopenias," "Muscle wasting," "Muscle atrophy," "Diabetes Mellitus," "Diabetes Insipidus" "Diet, Diabetic," "Prediabetic State" "Exercise Therapy" "Remedial Exercise," "Rehabilitation Exercises," "Physical Activities," "Aerobic Exercise," "Acute Exercise," "Isometric Exercise," "Exercise Training." The relevant references were tracked and noted.

#### Inclusion criteria and Exclusion criteria

Inclusion criteria: (1) Population: The study subjects included CKD patients with muscle atrophy or sarcopenia. (2) Intervention:Articles that employ exercise therapy as an intervention strategy are included, covering a range of modalities such as aerobic training, resistance training, and stretching exercises. Studies focusing on traditional physical therapy interventions, such as electrostimulation, and pharmacological treatments are excluded from this review. (3) Comparison: The control arm was subjected to conventional care, incorporating stretching routines or engaging in physical activities quantitatively inferior to the normative daily exertion levels. (4) Outcomes: Muscle Mass, Muscle Strength, Physical Performance, Biochemical Markers, Functional Status were measured at baseline and post-exercise. (5) Study: The literature categories comprised a variety of original research encompassing randomized controlled trials, quantitative studies, qualitative studies, and mixed studies.

Exclusion criteria: Reviews, conference abstracts, thematic summaries, protocols, duplicate publications, and full-text articles that were not accessible were excluded from the analysis. Literature that missed any discussion of the specific details regarding the execution, substance, or impact of exercise intervention in individuals afflicted with muscular atrophy due to chronic renal illness was likewise disregarded.

### Study selection

Two proficient researchers conducted an exhaustive review of titles and abstracts based on the inclusion and exclusion criteria, completing preliminary literature screening. Subsequently, they reviewed the complete text to make a final selection. The screening was conducted independently, and then the results were compared. The issue was referred to the research team for discussion in a dispute.Through open dialogue and a rigorous examination of the inclusion and exclusion criteria, we reconciled differing viewpoints and reached a mutually agreed-upon selection of literature. This process highlights the critical role of collaborative discussion and meticulous scrutiny in achieving a consensus on literature screening outcomes, ensuring the integrity and quality of our research methodology.

### Data extraction and synthesis

Two researchers independently extracted all the information from the included literature as follows: (1) authors, research type, country and year of publication, and sample size; (2) intervention method, intervention content and type, intervention duration of the experimental group and the control group; (3) outcome indicators, and measurement content of outcome indicators.

## Results

### Study characteristics

Following the initial retrieval of 875 articles, a final selection of 20 articles was made [10–29]. Figure 1 depicts the specific screening process. These included studies were conducted in China (*n* = 9), United Kingdom(*n* = 3), Brazil (*n* = 2), Australia (*n* = 1), Germany (*n* = 1), Italy (*n* = 1), South Korea (= 1), Sweden (*n* = 1), and the USA (*n* = 1). Regarding the publication years of the studies included in this article, the distribution is as follows: 2023 (*n* = 3), 2022 (*n* = 4), 2021 (*n* = 4), 2019 (*n* = 3), 2018 (*n* = 1), 2016 (*n* = 1), 2015 (*n* = 1), 2014 (*n* = 2), and 2007 (*n* = 1). The distribution of the studies by year and geographic location is presented in the supplementary figure. Among the 20 pieces of literature included in this study, 14 literatures described RCTs [12–27] and six literatures are quantitative non-random studies [10, 11, 13, 19, 22, 29]. Table 1 lists the characteristics of the included literature and is discussed below.

**Table 1:** Characteristics of the included studies (*n* = 20)

Author & Year	Method	Subjects		Therapies	
		EG	CG	EG	CG
Anding et al 2015 [10]	quantitative non-randomized study	46	-	Resistance training: RPE13 ~ 14, 2 times/week, 60 min/time	-
Bae et al 2014 [11]	quantitative non-randomized study	10	-	Warm-up: 5 min Aerobic training: 30 min, pedaling speed at about 35 rpm, RPE10 ~ 13 Cool-down and stretching	-
Kirkmanet al 2014 [12]	RCT	19	8	Resistance training: the maximum resistance is 200 kg, 8 ~ 10 times per group, complete 3 groups	Progressive stretching with resistance bands
Zhang Bo et al 2019 [13]	quantitative non-randomized study	11	-	Resistance training: progressive resistance training using elastic bands and hand-held weights, 20 ~ 30 min, 3 times per week Aerobic exercise: Treadmill training, 30 ~ 50 min, low intensity is 25% ~ 44% VO_2__max_, medium intensity is 45% ~ 59% VO_2__max_	-
Gadelhaet al 2021 [14]	RCT	57	50	Resistance training: chest compression, deep squats, rowing, knee and hip flexion and extension, shoulder compression, hip bridge, biceps curl, elbow extension, sitting leg lift; 3 times per week. Training intensity: RPE is 5–6 for the first 12 weeks and 7–8 for the last 12 weeks	Care as usual
Myers et al 2021 [15]	RCT	13	15	Aerobic combined with resistance training, with intensity of RPE12 ~ 14, reaching 70% ~ 80% of maximum heart rate	Care as usual
Chen Guanjie et al 2022 [16]	RCT	28	28	Resistance training: centripetal and centrifugal exercises using dumbbells, elastic bands, sandbags, weight-bearing ankle sheaths, and leg compression devices; Aerobic training: cycling in the air or using stationary bicycles, 10 to 15 times per action, total time 30 to 60 min, training intensity of 55% to 70% maximum heart rate, 2–3 times per week	Care as usual
Lopes et al 2019 [17]	RCT	30	20	Resistance training: knee and hip flexion and extension, sitting leg lift, leg compression. 20–40 min per time, 3 times per week	Stretching training
Zhou et al 2021 [18]	RCT	53	59	Resistance training: quadriceps stretch, deep squat, biceps curl, and pull-up. 2–3 times per set, 10 repetitions, 30 min per session. The frequency of exercise is 3 times per week, and the intensity of each training is RPE13-17	Balance training: Stand on a balance board or plank
Li Ming.et al 2022 [19]	quantitative non-randomized study	42	A:40B:38	Personalized resistance training combined with nutrition guidance using the HAPA model	Control group A: received nutritional guidance and took 250 ml of the enteral nutrition preparation orally for nutritional supplementation Control group B: personalized resistance training based on HAPA model, including upper limb elastic ball exercise, lower limb ankle weight loading, and straight leg raising. The intensity was 60% to 80% of the maximum heart rate, 3 sets/day, 1–2 h/time, 3 times/week
Cheema et al 2007 [20]	RCT	20	19	Resistance training: upper and lower limb exercises, 8 repetitions per group, totaling 10 groups. The intensity of each exercise is RPE15 ~ 17, and the exercise is performed three times per week	Care as usual, followed by resistance training after 12 weeks
Geneen et al 2022 [21]	RCT	10	7	Resistance training: leg press, knee extension, hamstring flexor and calf raises. Training intensity is 80% of 1-RM. Each set contains 8 repetitions, 3 sets per session, 3 times per week	Same as the experimental group, once a week
Fang Meng et al 2023 [22]	quantitative non-randomized study	29	30	Combined aerobic and resistance training: Aerobic training includes pedaling a bicycle at an intensity of RPE11 ~ 12, three times per week. Resistance training involves using elastic balls to perform gripping exercises ten times per group, a total of ten groups. Training involving flexion and extension of the knee and hip joints are performed using elastic straps, three times per week	Care as usual
Pomidori et al 2016 [23]	RCT	22	20	Aerobic training: Walking training, increasing the speed when the patient can tolerate. Training twice a day for ten minutes each time	Care as usual
Watson et al 2018 [24]	RCT	20	21	Aerobic training combined with resistance training:Aerobic training includes treadmill, cycling or rowing at an intensity of RPE12 ~ 14 for 30 min each time, three times a week; resistance training includes leg stretching and leg pressing, with the training load being 70% of the maximum single repetition, 12–15 repetitions per group, and three groups in total	The control group only performed aerobic training in the same way as the experimental group
Han Mei et al 2023 [25]	RCT	41	41	Resistance training: Three times per week, with a training cycle of four weeks	Care as usual
Peng Dandan et al 2023 [26]	RCT	43	43	Resistance training: lower limb ankle weight bearing and upper limb elastic ball movement, elastic ball grasping exercise. The exercise intensity starts from RPE 9 ~ 11 and gradually increases in moderate intensity RPE 12 ~ 13. Each time is 30 ~ 60 min, three times a week	Patients can choose to walk (80 ~ 90 steps/minute), jog (7 ~ 8 km/h), go up and down stairs (10 ~ 20 steps/minute)
He Tonglin et al 2019 [27]	RCT	21	21	Resistance training: The initial training intensity is 15-25RM, gradually increasing to 6 ~ 12RM, 60 min per time, 2–3 times per week	Walking, jogging, going up and down stairs. Perform muscle stretching after training
He Huixia et al 2022 [28]	RCT	90	90	Resistance training: Start with a physical load of 15–25 RM and gradually increase to 6 ~ 12RM, 40 min per session, 2–3 times per week Aerobic training: Warm-up and cool-down exercises, each performed for 10 min	Conduct non-resistance training such as walking (average 80–100 steps per minute), jogging (7–8 km/h), and walking up and down stairs (15–20 steps per minute). Exercise for a total of 1 h, 2–3 times per week
Wu Qian et al 2021 [29]	quantitative non-randomized study	58	57	Aerobic training: Perform Baduanjin training before hemodialysis, 20–30 min each time, 3 times a week	Care as usual
Intervention Duration				Outcome Measures & Measurement Tools	Main Results
12 months				Physical Performance:*6MWT* Patient-Reported Outcomes:*QoL*	High and moderate adherence groups showed notable enhancements in exercise capacity, strength, and several quality of life subscales
12 weeks				Physical Performance:*6MWT, Resting metabolic rate, Lactate threshold* Body composition:*Weight, Muscle mass, Body fat mass, Fat percentage, Body mass index* Skeletal Muscle Status:*Skeletal Muscle Mass Index, Right knee extension and flexion, Left knee extension and flexion* Patient-Reported Outcomes:*Quality of Life Questionnaire* Pulmonary function:*VO2max*	After 12 weeks of aerobic training, chronic kidney disease patients undergoing hemodialysis experienced a significant increase in the distance covered in the six-minute walk test (*P*< 0.05)
12 weeks				Physical Performance: *6MWT, Sit to stand, 8-ft get up and go* Skeletal Muscle Status:*Muscle volume, Knee extensor strength*	A 12-week high-intensity progressive resistance exercise training (PRET) regimen significantly increased thigh muscle volume and strength in hemodialysis patients
12 weeks				Physical Performance:*6MWT, Seat forward test, Eye-opening one-legged standing balance test* Skeletal Muscle Status:*Appendicular skeletal muscle mass, Grip strength Index* Laboratory examination:*Albumin, Renal profile* , *Urine albumin High-Density Lipoprotein and Low-Density Lipoprotein* Pulmonary function:*VO2 *_*max*_	Structured exercise training significantly improves muscle mass, muscle strength, and motor function, as evidenced by improved ASMI, grip strength index, 6-m walk speed, VO2peak, one-leg standing time, and reach in seat distance (*P* < 0.05), highlighting the potential of exercise interventions in this population
24 weeks				Physical Performance:*Time up-and-go, 6MWT* Body composition:*Body mass, BMI, Free fat mass,Body fat* Skeletal Muscle Status:*Handgrip* Laboratory examination: *Albumin, Potassium, Phosphorous,Calcium* Patient-Reported Outcomes:*Pain perception*	Findings revealed that resistance training before dialysis sessions significantly improved iron status, reduced ferritin and hepcidin levels, and favorably modulated inflammatory markers (TNFα, IL-6 decrease; IL-10 increase)
12 weeks				Physical Performance:*1-min sit-to-stand test*, *6MWT* Body composition:*Total leg mass,Total body mass,Total body fat* Skeletal Muscle Status:*Hand grip, Upper body strength, Lower body strength* Pulmonary function: *Forced Vital Capacity, Forced Expiratory Volume in One Second*	Findings revealed that the 12-week home-based exercise program significantly improved physical function, exercise capacity, and certain aspects of health-related quality of life (HRQL) among elderly patients undergoing maintenance hemodialysis
12–18 weeks				Physical Performance:*6MWT* Body composition:*BMI* Skeletal Muscle Status:*Hand grip, upper arm muscular circumference, calf circumference* Patient-Reported Outcomes: *Short Physical Performance Battery*	The study found that implementing an exercise program during dialysis significantly reduced the prevalence of sarcopenia in the experimental group compared to the control group (P = 0.014). Improvements were observed in grip strength, usual gait speed, calf circumference, and Short Physical Performance Battery (SPPB) scores (P < 0.05 for all)
12 weeks				Body composition:*Body mass, Lean mass, Fat mass* Laboratory examination:*IL-6, IL-10, TNF-a* Skeletal Muscle Status:*Hand grip* Patient-Reported Outcomes: *Short Physical Performance Battery*	The study observed that a 12-week home-based exercise regimen led to modest enhancements in physical function, exercise capacity, responses to exercise, pulmonary function, and health-related quality of life
12 months				Physical Performance: *6MWT, Functional reach* Body composition*: BMI,Arm lean mass, Leg lean mass, Trunk lean mass* Skeletal Muscle Status: *Hand grip, isometric quadriceps strength* Laboratory examination: *mGFR* Patient-Reported Outcomes: *Berg’s balance test*	The findings indicate that exercise training effectively prevents sarcopenia and maintains muscle mass. Despite significant increases in plasma myostatin levels in both exercise groups, these changes were not negatively associated with muscle mass or physical performance outcomes
6 months				Physical Performance: *Time up-and-go, stand up and sit test* Skeletal Muscle Status:*Hand grip* Laboratory examination:*Albumin, Transferrin, Hemoglobin* Patient-Reported Outcomes:*SARC-F, Barthel Index*	The findings demonstrated that the combination of nutritional guidance and personalized resistance exercise significantly enhanced serum nutritional parameters and quality of survival in MHD patients with sarcopenia. The intervention led to improvements in serum hemoglobin, prealbumin, and transferrin levels
24 weeks				Physical Performance: *6MWT* Body composition: *Body weight* Skeletal Muscle Status: *Muscle cross-sectional area, Muscle attenuation,Knee extension strength, Hip abduction strength,Triceps strength, Specific tension* Laboratory examination:*Log C-reactive protein*	The study found that prolonged intradialytic PRT led to an increase in muscle cross-sectional area and improvements in muscular strength and exercise capacity
12 weeks				Physical Performance:*6MWT, stand up and sit test, Physical Activity Recall* Body composition: *Weight, BMI, Fat mass* Skeletal Muscle Status: *Knee extension peak force 45, Leg press peak force,Total muscle depth, anatomical cross-sectional area* Patient-Reported Outcomes: *Leicester Uraemic Symptom Score*	The study revealed that both low (once a week) and higher frequency (three times a week) resistance training over 12 weeks significantly improved vastus lateralis cross-sectional area, pennation angle, muscle strength, and physical function in stage-3 CKD patients. The higher frequency group showed greater anatomical and physiological muscle adaptations, yet improvements in strength and function were comparable between frequencies
12 weeks				Body composition: *BMI, visceral adipose tissue area* Skeletal Muscle Status: *Total Muscle Mass, Hand grip* Laboratory examination: *Albumin, Log C-reactive protein, Hemoglobin*	The study demonstrated that aerobic combined with resistance exercises significantly improved muscle strength, nutritional status, and quality of life in elderly patients undergoing maintenance hemodialysis with obesity-related sarcopenia. These interventions led to significant reductions in body fat percentage and visceral fat area, alongside notable increases in grip strength, serum albumin levels, and overall life quality scores
6 months				Physical Performance: *6MWT* Pulmonary function: *Forced Expiratory Volume in One Second, Vital Capacity, Maximum Voluntary Ventilation, Maximal Inspiratory Pressure*	The study investigated the impact of a 6-month moderate-intensity exercise program on respiratory muscle strength in dialysis patients, comparing trained versus untrained groups. The results indicated that the exercise program improved physical capacity and preserved respiratory muscle function in the trained group, contrasting with the deterioration observed in the untrained group
12 weeks				Physical Performance: *Incremental shuttle walk test* Skeletal Muscle Status: *Muscle volume* Body composition: *Weight* Pulmonary function: *VO2*_*max*_	The study revealed that combining aerobic and resistance exercise for 12 weeks produced greater increases in muscle mass and strength compared to aerobic exercise alone in patients with chronic kidney disease not on dialysis. Significant improvements were observed in knee extensor strength and quadriceps volume
12 weeks				Skeletal Muscle Status: *MRC muscle strength scoring system* Patient-Reported Outcomes: *Sarcopenia quality of life,* *SarQoL*	The study found that implementing periodic resistance training alongside a reasonable diet significantly enhanced muscle strength and overall quality of life in hemodialysis patients suffering from sarcopenia. This intervention showed notable improvements in both upper and lower limb muscle strength grading scores and scores across various domains of the Sarcopenia Quality of Life (SarQoL) questionnaire
12 weeks				Physical Performance: *6MWT* Skeletal Muscle Status: *Hand grip, skeletal muscle mass index, Upper arm muscular circumference, calf circumference* Patient-Reported Outcomes: *Short Physical Performance Battery*	The study found that implementing a 12-week progressive resistance exercise (PRE) program significantly improved handgrip strength (HGS), daily walking speed, and SPPB (Short Physical Performance Battery) scores. Moreover, skeletal muscle mass index (SMI), arm circumference, and calf circumference were significantly higher in the PRE group compared to the control group
8–12 weeks				Skeletal Muscle Status: *Skeleton appendiculare* Patient-Reported Outcomes: *Activities of Daily Living*	The study demonstrated that exercise rehabilitation therapy significantly improved muscle mass and strength in patients with uremic sarcopenia, thereby offering notable therapeutic and preventive benefits for uremic sarcopenia. The therapy led to statistically significant increases in skeletal muscle mass at 8 and 12 weeks, as well as higher activities of daily living (ADL) scores in the treatment group compared to the control group
12 months				Skeletal Muscle Status: *Skeleton appendiculare* Patient-Reported Outcomes: *Activities of Daily Living*	The study demonstrated that exercise rehabilitation therapy significantly improved skeletal muscle mass and the ability to perform activities of daily living (ADL) in patients with uremic sarcopenia. Notably, both male and female patients in the intervention group showed significant increases in skeletal muscle mass and ADL scores after 6 and 12 months of rehabilitation, compared to the control group
12 weeks				Physical Performance: *6MWT* Skeletal Muscle Status: *Hand grip, Skeletal muscle mass index* Patient-Reported Outcomes:* International physical activity questionnaire short form*	The study found that practicing Baduanjin exercise prior to hemodialysis sessions significantly improved hand grip strength, daily walking speed, and physical activity levels, while reducing fatigue as measured by the revised Piper Fatigue Scale (RPFS), compared to a control group receiving standard care and exercise guidance

*HDL* High-Density Lipoprotein, *LDL* Low-Density Lipoprotein, *EG*  Experimental Group, *CG* Control Group

### Types of Exercise Intervention Programs

The exercise intervention scheme mainly includes resistance exercise [12–28], aerobic exercise [23, 29], and aerobic exercise combined with resistance exercise [13, 15, 22, 24]. (1) Currently, resistance training is the most prevalent form of exercise intervention. The common resistance exercise intervention scheme involves strength training of the upper and lower limbs. Upper limb strength training includes biceps curl with dumbbells, elastic ball movement on the non-internal fistula side, pull-ups, and chest and shoulder compression. However, lower limb strength training focuses on the muscle groups around the knee joint, including lunge, squat, sitting leg raising, knee flexion and extension, and quadriceps strength training with elastic belts. Furthermore, there are resistance exercises for the hip joint, such as leg compression and hip flexion. (2) The aerobic exercise intervention scheme primarily consists of several modalities, such as bicycle modalities [11, 16], treadmill exercise [13], Baduanjin [29], and steady-state walking [23]. In this context, Luca et al. [23] used steady-state walking as an intervention plan for aerobic exercise and examined the impact of this simple aerobic exercise on the subjects. (3) The intervention program mostly incorporates resistance exercise with low-to-moderate intensity aerobic exercise. The three most prevalent forms of aerobic exercise are cycling, treadmill, and rowing. Liming et al. [19] used psychological theory to guide patients in resistance exercise, helping patients develop healthy behaviors through the healthy action process orientation model.

### Intensity, frequency, and duration of exercise intervention

The intervention intensity and frequency varied among the studies. (1) Exercise intensity: Eight studies [10–12, 18, 19, 22, 24, 26] set the exercise intensity according to the scores of the subjects' perceived exertion (RPE). The intensity target of exercise training was established in two studies [14, 26] based on the patient's capacity to tolerate the quantity of exercise. The training intensity progressively escalated until the patients reached the optimal tolerance level. Two studies [21, 27] assessed the exercise intensity of the subjects based on maximum repetitions (RM) and the percentage of maximum loading (1RM) of the exercise load. Four studies [13, 15, 16, 19] set the exercise intensity based on the subjects' cardiopulmonary function indexes. This was achieved by measuring the heart rate of the patients during exercise as a reference, setting the appropriate exercise intensity after evaluating the heart rate at the maximum exercise intensity they could tolerate. Zhang Bo et al. [13] divided the exercise training into low intensity (25% ~ 44% VO_2__max_) and moderate intensity (45% ~ 59% VO_2__max_) based on the subjects' peak oxygen uptake (VO_2__max_). There was a gradual transition from low to moderate intensity during the intervention. This. (2) Exercise frequency: The intervention frequency of each study varied from 2 to 3 times per week, while the duration of intervention was typically set based on the subjects' tolerance level and training objectives. (3) Duration of exercise intervention: There are a total of 13 studies in which the intervention period spans 12 weeks [11–13, 15–17, 21–27, 29], two studies have an intervention duration of 24 weeks [14, 19], three studies have an intervention duration of 12 months [10, 18, 28], and two studies have an intervention duration of 6 months [19, 23].

### Outcome indicators and measurement tools

Outcome indicators included functional tests, body composition, strength, laboratory examination, cardiopulmonary function, and strength, and functional evaluations. The principal physical activity assessments comprised a walk test, sit-to-stand test, functional reach, balance test, SPPB score, and physical activity recall. The measurement of body composition included weight, body mass index, body fat, and lean body mass. Skeletal muscle mass was assessed using dual-energy X-ray absorptiometry, anthropometry, and muscle ultrasound. Laboratory examination indicators included inflammatory markers, renal profile, and body fat. Cardiopulmonary function indicators examined forced expiratory volume in one second (FEV-1), vital capacity, forced vital capacity (FVC), maximum inspiratory pressure (MIP), and peak oxygen uptake (VO_2__max_). In terms of patient-reported outcomes, the primary measurement tools encompassed the Quality-of-Life Questionnaire, Pain Scale, Leicester Uremic Symptom Score, Sarcopenia Quality of Life Scale, Activities of Daily Living Scale, Barthel Index, Kidney Disease Quality of Life Scale, Dialysis Patient Quality of Life Scale, and Revised Piper Fatigue Scale.

### Effects of Exercise Intervention

(1) Effects of resistance exercise intervention: The exercise intervention schemes for muscle atrophy in CKD patients in the literature included different levels of resistance exercise. A total of ten studies have documented that resistance training protocols yielded a positive impact on several aspects of the subjects' physical function [10, 11, 13, 16, 29], muscle strength [10, 13, 16, 22, 26–29], daily living ability [10, 13, 27] and renal function [19, 22]. Danielle and colleagues [12] found that a 12-week program of high-intensity progressive resistance exercise training (PRET) for hemodialysis patients led to a statistically and clinically significant increase in thigh muscle volume, with a mean difference of 193 cm^3^ (95% CI: 63 to 324 cm^3^; *P* = 0.007) compared to the control group. However, it did not significantly enhance the subjects' physical activity capacity or overall quality of life. (2) Aerobic exercise intervention effect: Eleven studies [11, 13, 15, 16, 22–28] reported the effects of different levels of aerobic exercise on subjects. Since low-intensity aerobic exercise was difficult to improve muscle strength [30] significantly, it was mostly used as an exercise intervention program for the control group [11, 13, 16, 22, 23, 26–28]. Furthermore, other studies showed positive impacts of aerobic exercise training interventions on the subjects. For example, Wu Qian et al. [29] found significant improvements in the intervention group's handgrip strength, daily walking speed, and physical activity level compared to the control group, with the revised Piper fatigue scale (RPFS) scores also being significantly lower (indicating less fatigue) in the intervention group. These differences were statistically significant (*P* < 0.05). (3) Effects of aerobic exercise combined with resistance exercise intervention: Four literatures [13, 15, 22, 24] incorporated a combination of aerobic exercise and resistance training in their physical exercise intervention program. Emma et al. [24] showed that combined resistance and aerobic training were more beneficial than aerobic training alone. Zhang Bo et al. [13] demonstrated that after 12 weeks of low and medium intensity aerobic combined with resistance exercise, elderly patients with chronic kidney disease complicated with sarcopenia showed significant improvements in appendicular skeletal muscle mass index (ASMI), grip strength index, 6-m walking speed, peak oxygen uptake (VO_2__max_), one-leg standing time, and reach in seat distance, with p-values indicating statistical significance (*P* < 0.05) for these improvements. This indicates that the exercise intervention was effective in enhancing muscle mass, muscle strength, and motor function without adversely affecting renal function.

## Discussion

This study demonstrates that exercise intervention for muscle atrophy in individuals with CKD includes different modalities, mostly resistance training and a combination of aerobic exercise. Guiding patients regarding resistance training can facilitate the anabolism and metabolism of skeletal muscle, enhancing muscle quality, improving muscle strength, and effectively enhancing patients' overall quality of life [31]. Exercise intervention schemes for CKD patients are often carried out in various forms of exercise combinations. Researchers often use aerobic exercise as an important auxiliary intervention method that benefits cardiovascular health. It improves the heart and lung function of the subjects, enhances their endurance, and facilitates the development of resistance training.

There are significant differences among CKD patients at different stages, so we should comprehensively evaluate the physiological function and tolerance of CKD patients and develop individualized exercise intervention programs for patients [7]. This study showed that the exercise intervention program includes detailed pre-exercise examinations, such as preliminarily assessing the subjects’ muscle strength and cardiopulmonary function status, determining whether they can tolerate exercise intervention, and developing personalized exercise prescriptions. Jonathan [15] and colleagues completed detailed medical examinations before the intervention, measuring the subjects' cardiopulmonary function and upper and lower limb strength. Results indicated that the design of the exercise intervention program was personalized. This study showed that the exercise form of CKD patients is not a single repeat of a certain type of exercise but a combination of various exercise methods that can achieve the best effect. Despite numerous RCTs reported that resistance exercise could significantly improve muscle strength in patients with CKD [32], a meta-analysis [33] has revealed that the use of progressive resistance exercise as the sole intervention did not result in a significant improvement in the 6-min walk test, which serves as a proxy indicator of cardiopulmonary function. It indicates that resistance training alone makes it difficult to improve the cardiopulmonary function of subjects significantly. As resistance exercise training necessitates a minimum level of cardiopulmonary function as a foundation, it also imposes specific cardiopulmonary function requirements on the subjects. Low-intensity, long-duration aerobic exercise has been shown to enhance the cardiopulmonary function and exercise capacity of patients with chronic kidney disease [34]. Therefore, clinical researchers frequently combine two forms of exercise to enhance patients' quality of life by enhancing their cardiopulmonary function and muscle strength. It demonstrates the comprehensive nature of exercise intervention program design. Universality: For patients with non-maintenance dialysis CKD, regular visits to the hospital for dialysis treatment means that they cannot receive the supervision of clinical researchers throughout the process. They also face problems such as a lack of exercise equipment and professional guidance. Emma et al. [24] found that patients considered frequent visits to the hospital as a major obstacle to participating in the study. So, future research needs to design simple and feasible home training programs for different patient situations and provide feedback when patients come to the hospital for dialysis. Luca’s [23] exercise training program was designed and organized by the hospital and ultimately completed by patients at home. It improved patients' physical function significantly after six months of intervention with low-intensity exercise.

The results of this study show that exercise intervention can improve physical function, activity ability, muscle atrophy, and quality of life in patients with CKD. This viewpoint is also supported by Vanden et al.'s systematic review of exercise intervention in CKD patients [35]. Furthermore, a cross-sectional study [36] also suggested that clinical medical staff should actively implement exercise interventions for CKD patients to address adverse outcomes such as muscle atrophy and decreased quality of life caused by long-term catabolism and dialysis treatment. Although exercise intervention positively impacts CKD patients, the current studies are mainly small-sample randomized controlled trials with low quality. Therefore, larger-sample randomized controlled trials are needed to evaluate the impact of exercise intervention on muscle atrophy in CKD patients more accurately. Notably, the frequency and duration of exercise intervention are not positively correlated with the intervention effect. Excessive exercise intensity may lead to lactic acidosis in patients, and severe cases may cause acute renal failure secondary to rhabdomyolysis [37]. Therefore, exercise frequency must be determined by the patient's condition; high-intensity training should not be pursued indiscriminately. Furthermore, cardiac function in CKD patients must be matched with the intensity of exercise. A systematic review have shown that high-intensity interval exercise with passive recovery leads to a greater increase in cardiac troponin T levels compared to moderate-intensity continuous exercise [38]. To minimize cardiovascular risk during exercise, exercise prescriptions should be tailored based on individual circumstances.A unified standard regarding the exercise frequency and intervention cycle of CKD patients does not exist. The guidelines indicate that the optimal resistance exercise frequency is 2 − 3 times per week, with an interval of 24 − 48 h each time, which is consistent with the results shown in this study [39]. In the literature included in this study, only seven articles [12, 16, 17, 19, 25, 26, 29] mentioned the safety of exercise intervention, and 3 of them [12, 17, 19] reported adverse events. Danielle et al. [12] reported muscle soreness and laceration of the back wound, indicating that there is a lack of comprehensive reports regarding the safety of exercise intervention trials in CKD patients. Some researchers failed to fully consider the safety of exercise intervention based on individual differences among subjects.

Integrating insights from the studies on exercise's impact on CKD, sarcopenia in hemodialysis patients, and diabetes management through physical activity, the future perspective emphasizes a holistic approach to CKD treatment. This approach advocates for the incorporation of tailored exercise programs to address the multifaceted challenges of CKD. A study elucidates how diverse exercise modalities can significantly impact blood glucose levels, emphasizing the importance of exercise in the management of diabetes, particularly in patients with CKD. It underscores the role of physical activity in enhancing insulin sensitivity and glycemic control, offering a non-pharmacological strategy to mitigate diabetes-related complications in CKD patients [40]. Additionally, exercise has beneficial effects on mitochondrial function and diabetes management. The research emphasizes that various exercise modalities, such as resistance and endurance training, can enhance mitochondrial density, dynamics, and oxidative capacity in muscle tissues. These improvements aid in boosting glucose metabolism and insulin sensitivity, which are crucial for diabetes control. The findings indicate that structured exercise programs are an indispensable component in addressing mitochondrial dysfunction and managing diabetes, especially in populations with chronic conditions like CKD [41]. Moreover, research has identified the efficacy of exercise in alleviating fatigue among hemodialysis patients, highlighting the potential of structured exercise programs to enhance energy levels and overall health in this demographic, regular physical activity could be an essential component of comprehensive care for hemodialysis patients, thus improving their quality of life and physical function [42]. The envisioned future includes rigorous clinical trials to further validate exercise as a key adjunct therapy, potentially leading to standardized exercise recommendations that enhance patient outcomes and quality of life in CKD management.

## Strengths and limitations

The strengths of this scoping review include an extensive and repeated search of the literature to capture all relevant articles and limit emissions while strictly adhering to the PRISMA reporting guidelines. Unpublished dissertations are more likely to report uncertain or negative results, which may produce a reverse publication bias and be excluded from this study. The literature included in this study lacks large-sample, multi-center, randomized controlled studies, and the methodological rigor and data quality of the included studies have not been systematically evaluated.

## Conclusion

Patients with CKD face many complex issues that seriously affect their quality of life. For them, long-term dialysis treatment has produced negligible side effects, and exercise therapy is needed to improve their quality of life. Exercise intervention can improve the muscle strength, physical function, and quality of life of patients with chronic nephritis muscle atrophy, which is an effective intervention method. We are still waiting to find its potential positive effects on CKD patients. In future nursing work, we should evaluate the physiological function status of CKD patients, formulate safe and effective exercise intervention plans, and intervene early in their muscle atrophy symptoms to promote their rehabilitation and improve their clinical outcomes.

### Supplementary Information

**Supplementary Material 1.**

**Supplementary Material 2.**

**Supplementary Material 3.**

**Supplementary Material 4.**

## Data Availability

All data generated or analysed during this study are included in supplementary information files.
