# Supplementary material for: Application of exercise therapy in patients with chronic kidney disease-induced muscle atrophy: a scoping review
Source: BMC Sports Sci Med Rehabil. 2024 Apr 30;16:100. doi: 10.1186/s13102-024-00876-8 (PMC11061900; doi:10.1186/s13102-024-00876-8)
Supplement: Supplementary file 1 — Supplementary Material 1. [file 13102_2024_876_MOESM1_ESM.docx]

| **Database searched** | **Search terms** |
| --- | --- |
| **PubMed** | ("renal insufficiency, chronic" OR "chronic renal insufficiencies" OR "renal insufficiencies chronic" OR "chronic renal insufficiency" OR "kidney insufficiency chronic" OR "chronic kidney insufficiency" OR "Kidney insufficiencies, chronic" OR "chronic kidney diseases" OR "chronic kidney disease" OR "disease chronic kidney" OR "diseases chronic kidney" OR "kidney disease chronic" OR "kidney diseases chronic" OR "chronic renal diseases" OR "chronic renal disease" OR "disease chronic renal" OR "diseases chronic renal" OR "renal disease chronic" OR "renal diseases chronic")  **AND**  ("Sarcopenia" OR "Sarcopenias" OR "muscle wasting" OR "muscle atrophy")  **AND**  ("Exercise Therapy" OR "Remedial Exercise" OR "Exercise, Remedial" OR "Exercises, Remedial" OR "Remedial Exercises" OR "Therapy, Exercise" OR "Exercise Therapies" OR "Therapies, Exercise" OR "Rehabilitation Exercise" OR "Exercise, Rehabilitation" OR "Exercises, Rehabilitation" OR "Rehabilitation Exercises" OR "Physical Activity" OR "Activities, Physical" OR "Activity, Physical" OR "Physical Activities" OR "Exercise, Physical" OR "Exercises, Physical" OR "Physical Exercise" OR "Physical Exercises" OR "Acute Exercises" OR "Exercise, Acute" OR "Exercises, Acute" OR "Exercise, Isometric" OR "Exercises, Isometric" OR "Isometric Exercises" OR "Isometric Exercise" OR "Exercise, Aerobic" OR "Aerobic Exercise" OR "Aerobic Exercises" OR "Exercises, Aerobic" OR "Exercise Training" OR "Exercise Trainings" OR "Training, Exercise" OR "Trainings, Exercise") |
| **Cochrane Library** | MeSH descriptor: [Renal Insufficiency, Chronic] explode all trees  (chronic renal insufficiencies):ti,ab,kw OR (renal insufficiencies chronic):ti,ab,kw OR(chronic renal insufficiency):ti,ab,kw OR(kidney insufficiency chronic):ti,ab,kw OR(chronic kidney insufficiency):ti,ab,kw OR(Kidney insufficiencies, chronic):ti,ab,kw OR(chronic kidney diseases):ti,ab,kw OR(chronic kidney disease):ti,ab,kw OR(disease chronic kidney):ti,ab,kw OR(diseases chronic kidney):ti,ab,kw OR(kidney disease chronic):ti,ab,kw OR(kidney diseases chronic):ti,ab,kw OR(chronic renal diseases):ti,ab,kw OR(chronic renal disease):ti,ab,kw OR(disease chronic renal):ti,ab,kw OR(diseases chronic renal):ti,ab,kw OR(renal disease chronic):ti,ab,kw OR(renal diseases chronic):ti,ab,kw  MeSH descriptor: [Sarcopenia] explode all trees  (Sarcopenias):ti,ab,kw OR (muscle wasting):ti,ab,kw OR (muscle atrophy):ti,ab,kw  MeSH descriptor: [Exercise Therapy] explode all trees  (Remedial Exercise):ti,ab,kw OR (Exercise, Remedial):ti,ab,kw OR (Exercises, Remedial):ti,ab,kw OR (Remedial Exercises):ti,ab,kw OR (Therapy, Exercise):ti,ab,kw OR (Exercise Therapies):ti,ab,kw OR (Therapies, Exercise):ti,ab,kw OR (Rehabilitation Exercise):ti,ab,kw OR (Exercise, Rehabilitation):ti,ab,kw OR (Exercises, Rehabilitation):ti,ab,kw OR (Rehabilitation Exercises):ti,ab,kw OR (Physical Activity):ti,ab,kw OR (Activities, Physical):ti,ab,kw OR (Activity, Physical):ti,ab,kw OR (Physical Activities):ti,ab,kw OR (Exercise, Physical):ti,ab,kw OR (Exercises, Physical):ti,ab,kw OR (Physical Exercise):ti,ab,kw OR (Physical Exercises):ti,ab,kw OR (Acute Exercises):ti,ab,kw OR (Exercise, Acute):ti,ab,kw OR (Exercises, Acute):ti,ab,kw OR (Exercise, Isometric):ti,ab,kw OR (Exercises, Isometric):ti,ab,kw OR (Isometric Exercises):ti,ab,kw OR (Isometric Exercise):ti,ab,kw OR (Exercise, Aerobic):ti,ab,kw OR (Aerobic Exercise):ti,ab,kw OR (Aerobic Exercises):ti,ab,kw OR (Exercises, Aerobic):ti,ab,kw OR (Exercise Training):ti,ab,kw OR (Exercise Trainings):ti,ab,kw OR (Training, Exercise):ti,ab,kw OR (Trainings, Exercise):ti,ab,kw OR (Training):ti,ab,kw OR (Exercise):ti,ab,kw |
| **Web of Science** | TS=(renal insufficiency, chronic or chronic renal insufficiencies or renal insufficiencies chronic or chronic renal insufficiency or kidney insufficiency chronic or chronic kidney insufficiency or Kidney insufficiencies, chronic or chronic kidney diseases or chronic kidney disease or disease chronic kidney or diseases chronic kidney or kidney disease chronic or kidney diseases chronic or chronic renal diseases or chronic renal disease or disease chronic renal or diseases chronic renal or renal disease chronic or renal diseases chronic)  TS=(Sarcopenia or Sarcopenias or muscle wasting or muscle atrophy)  TS=(Exercise Therapy or Remedial Exercise or Exercise, Remedial or Exercises, Remedial or Remedial Exercises or Therapy, Exercise or Exercise Therapies or Therapies, Exercise or Rehabilitation Exercise or Exercise, Rehabilitation or Exercises, Rehabilitation or Rehabilitation Exercises or Physical Activity or Activities, Physical or Activity, Physical or Physical Activities or Exercise, Physical or Exercises, Physical or Physical Exercise or Physical Exercises or Acute Exercises or Exercise, Acute or Exercises, Acute or Exercise, Isometric or Exercises, Isometric or Isometric Exercises or Isometric Exercise or Exercise, Aerobic or Aerobic Exercise or Aerobic Exercises or Exercises, Aerobic or Exercise Training or Exercise Trainings or Training, Exercise or Trainings, Exercise or Training or Exercise) |
| **ProQuest** | AB,TI("renal insufficiency, chronic" OR "chronic renal insufficiencies" OR "renal insufficiencies chronic" OR "chronic renal insufficiency" OR "kidney insufficiency chronic" OR "chronic kidney insufficiency" OR "Kidney insufficiencies, chronic" OR "chronic kidney diseases" OR "chronic kidney disease" OR "disease chronic kidney" OR "diseases chronic kidney" OR "kidney disease chronic" OR "kidney diseases chronic" OR "chronic renal diseases" OR "chronic renal disease" OR "disease chronic renal" OR "diseases chronic renal" OR "renal disease chronic" OR "renal diseases chronic" )  AB,TI("Sarcopenia" OR "Sarcopenias" OR "muscle wasting" OR "muscle atrophy")  AB,TI("Exercise Therapy" OR "Remedial Exercise" OR "Exercise, Remedial" OR "Exercises, Remedial" OR "Remedial Exercises" OR "Therapy, Exercise" OR "Exercise Therapies" OR "Therapies, Exercise" OR "Rehabilitation Exercise" OR "Exercise, Rehabilitation" OR "Exercises, Rehabilitation" OR "Rehabilitation Exercises" OR "Physical Activity" OR "Activities, Physical" OR "Activity, Physical" OR "Physical Activities" OR "Exercise, Physical" OR "Exercises, Physical" OR "Physical Exercise" OR "Physical Exercises" OR "Acute Exercises" OR "Exercise, Acute" OR "Exercises, Acute" OR "Exercise, Isometric" OR "Exercises, Isometric" OR "Isometric Exercises" OR "Isometric Exercise" OR "Exercise, Aerobic" OR "Aerobic Exercise" OR "Aerobic Exercises" OR "Exercises, Aerobic" OR "Exercise Training" OR "Exercise Trainings" OR "Training, Exercise" OR "Trainings, Exercise" OR "Training" OR "Exercise") |
| **Ovid** | (renal insufficiency, chronic or chronic renal insufficiencies or renal insufficiencies chronic or chronic renal insufficiency or kidney insufficiency chronic or chronic kidney insufficiency or Kidney insufficiencies, chronic or chronic kidney diseases or chronic kidney disease or disease chronic kidney or diseases chronic kidney or kidney disease chronic or kidney diseases chronic or chronic renal diseases or chronic renal disease or disease chronic renal or diseases chronic renal or renal disease chronic or renal diseases chronic).ti,ab,kw.  (Sarcopenia or Sarcopenias or muscle wasting or muscle atrophy).ti,ab,kw.  (Exercise Therapy or Remedial Exercise or Exercise, Remedial or Exercises, Remedial or Remedial Exercises or Therapy, Exercise or Exercise Therapies or Therapies, Exercise or Rehabilitation Exercise or Exercise, Rehabilitation or Exercises, Rehabilitation or Rehabilitation Exercises or Physical Activity or Activities, Physical or Activity, Physical or Physical Activities or Exercise, Physical or Exercises, Physical or Physical Exercise or Physical Exercises or Acute Exercises or Exercise, Acute or Exercises, Acute or Exercise, Isometric or Exercises, Isometric or Isometric Exercises or Isometric Exercise or Exercise, Aerobic or Aerobic Exercise or Aerobic Exercises or Exercises, Aerobic or Exercise Training or Exercise Trainings or Training, Exercise or Trainings, Exercise or Training or Exercise).ti,ab,kw. |
| **EMBASE** | 'renal insufficiency, chronic':ab,ti or 'chronic renal insufficiencies':ab,ti or 'renal insufficiencies chronic':ab,ti or 'chronic renal insufficiency':ab,ti or 'kidney insufficiency chronic':ab,ti or 'chronic kidney insufficiency':ab,ti or 'Kidney insufficiencies, chronic':ab,ti or 'chronic kidney diseases':ab,ti or 'chronic kidney disease':ab,ti or 'disease chronic kidney':ab,ti or 'diseases chronic kidney':ab,ti or 'kidney disease chronic':ab,ti or 'kidney diseases chronic':ab,ti or 'chronic renal diseases':ab,ti or 'chronic renal disease':ab,ti or 'disease chronic renal':ab,ti or 'diseases chronic renal':ab,ti or 'renal disease chronic':ab,ti or 'renal diseases chronic':ab,ti  'Sarcopenia':ab,ti or 'Sarcopenias':ab,ti or 'muscle wasting':ab,ti or 'muscle atrophy':ab,ti  'Exercise Therapy':ab,ti or 'Remedial Exercise':ab,ti or 'Exercise, Remedial':ab,ti or 'Exercises, Remedial':ab,ti or 'Remedial Exercises':ab,ti or 'Therapy, Exercise':ab,ti or 'Exercise Therapies':ab,ti or 'Therapies, Exercise':ab,ti or 'Rehabilitation Exercise':ab,ti or 'Exercise, Rehabilitation':ab,ti or 'Exercises, Rehabilitation':ab,ti or 'Rehabilitation Exercises':ab,ti or 'Physical Activity':ab,ti or 'Activities, Physical':ab,ti or 'Activity, Physical':ab,ti or 'Physical Activities':ab,ti or 'Exercise, Physical':ab,ti or 'Exercises, Physical':ab,ti or 'Physical Exercise':ab,ti or 'Physical Exercises':ab,ti or 'Acute Exercises':ab,ti or 'Exercise, Acute':ab,ti or 'Exercises, Acute':ab,ti or 'Exercise, Isometric':ab,ti or 'Exercises, Isometric':ab,ti or 'Isometric Exercises':ab,ti or 'Isometric Exercise':ab,ti or 'Exercise, Aerobic':ab,ti or 'Aerobic Exercise':ab,ti or 'Aerobic Exercises':ab,ti or 'Exercises, Aerobic':ab,ti or 'Exercise Training':ab,ti or 'Exercise Trainings':ab,ti or 'Training, Exercise':ab,ti or 'Trainings, Exercise':ab,ti or 'Training':ab,ti or 'Exercise':ab,ti |
