# Supplementary material for: Application of exercise therapy in patients with chronic kidney disease-induced muscle atrophy: a scoping review
Source: BMC Sports Sci Med Rehabil. 2024 Apr 30;16:100. doi: 10.1186/s13102-024-00876-8 (PMC11061900; doi:10.1186/s13102-024-00876-8)
Supplement: Supplementary file 3 — Supplementary Material 3. [file 13102_2024_876_MOESM3_ESM.docx]

Figure S2: Literature publications since 2007.
