# Supplementary figures and images for: Application of exercise therapy in patients with chronic kidney disease-induced muscle atrophy: a scoping review
Source: BMC Sports Sci Med Rehabil. 2024 Apr 30;16:100. doi: 10.1186/s13102-024-00876-8 (PMC11061900; doi:10.1186/s13102-024-00876-8)

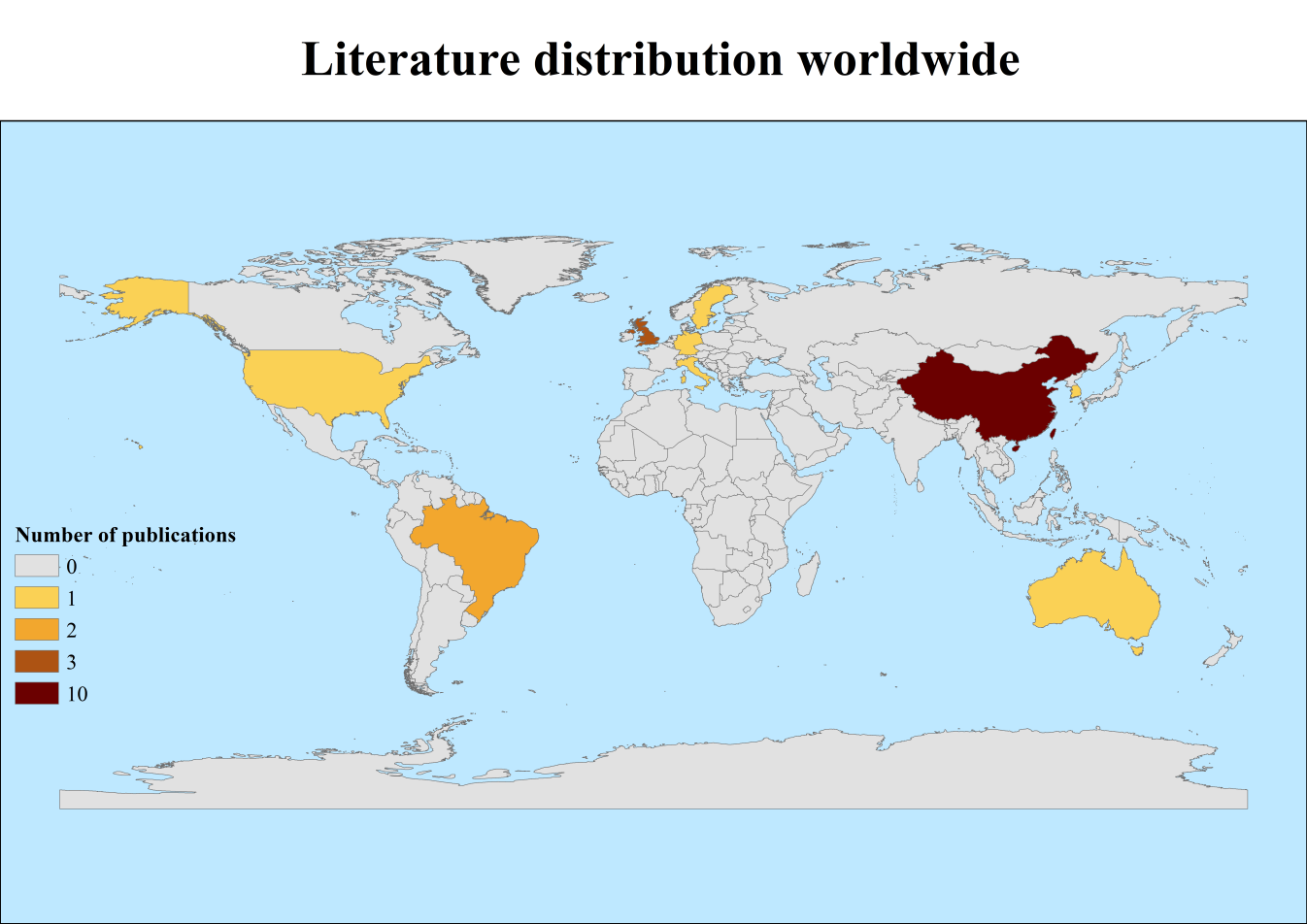


Figure S3:Literature distribution worldwide.

Supplement: Supplementary file 4 — Supplementary Material 4. [file 13102_2024_876_MOESM4_ESM.docx]
